# The Diversity of Coral Reefs: What Are We Missing?

**DOI:** 10.1371/journal.pone.0025026

**Published:** 2011-10-13

**Authors:** Laetitia Plaisance, M. Julian Caley, Russell E. Brainard, Nancy Knowlton

**Affiliations:** 1 Department of Invertebrate Zoology, National Museum of Natural History, Smithsonian Institution, Washington, D.C., United States of America; 2 Center for Marine Biodiversity and Conservation, Scripps Institution of Oceanography, University of California San Diego, La Jolla, California, United States of America; 3 Australian Institute of Marine Science, Townsville, Australia; 4 Coral Reef Ecosystem Division, Pacific Islands Fisheries Science Center, National Oceanic and Atmospheric Administration–Fisheries, Honolulu, Hawaii, United States of America; California Academy of Sciences, United States of America

## Abstract

Tropical reefs shelter one quarter to one third of all marine species but one third of the coral species that construct reefs are now at risk of extinction. Because traditional methods for assessing reef diversity are extremely time consuming, taxonomic expertise for many groups is lacking, and marine organisms are thought to be less vulnerable to extinction, most discussions of reef conservation focus on maintenance of ecosystem services rather than biodiversity loss. In this study involving the three major oceans with reef growth, we provide new biodiversity estimates based on quantitative sampling and DNA barcoding. We focus on crustaceans, which are the second most diverse group of marine metazoans. We show exceptionally high numbers of crustacean species associated with coral reefs relative to sampling effort (525 species from a combined, globally distributed sample area of 6.3 m^2^). The high prevalence of rare species (38% encountered only once), the low level of spatial overlap (81% found in only one locality) and the biogeographic patterns of diversity detected (Indo-West Pacific>Central Pacific>Caribbean) are consistent with results from traditional survey methods, making this approach a reliable and efficient method for assessing and monitoring biodiversity. The finding of such large numbers of species in a small total area suggests that coral reef diversity is seriously under-detected using traditional survey methods, and by implication, underestimated.

## Introduction

Reef species diversity has been estimated at ∼600,000 to more than 9 million species worldwide [1]–[3] This diversity is concentrated in the central Indo-Pacific [4] (the “Coral Triangle”), and decreases with increasing distance from the Indo-Australian archipelago. Traditionally, large and well-studied macrofauna, such as corals and fishes, have been used as surrogates in biodiversity assessments [5] because they are comparatively easy to census and taxonomically well known. However, these two groups represent just a tiny fraction of reef-associated diversity, and the use of a few groups as surrogates for biodiversity assessment may not capture patterns of diversity across all organisms [6], [7].

Reefs are also one of the most endangered habitats of the planet [8]. The loss of corals and the associated potential threat to biodiversity [9], [10] are well established, but we still remain largely ignorant of the details, and conservation priorities are often based on what we can measure. Providing a reliable method that estimates biodiversity across space and through time is essential for designing the specifics of marine protected areas and for monitoring their effectiveness. Inventory data on small organisms collected to assess coral reef diversity largely consist of taxonomic identifications of collected material through non-standardized sampling strategies. The limitations of these methods are obvious: the results are not comparable from site to site because the sampling effort is not quantifiable, the number of specimens processed is limited by a very time-consuming approach that depends on the availability of taxonomic expertise, and cryptic species are not detected leading to underestimation of the real biodiversity.

**Figure 1:**
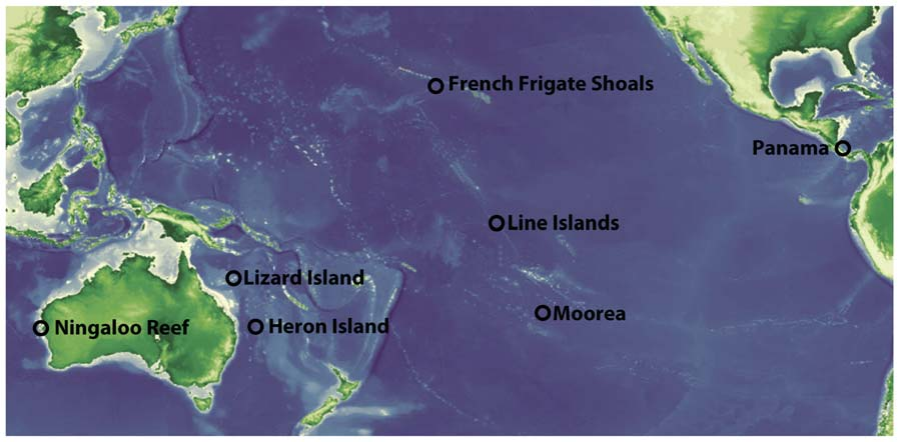
Sampling localities in the Indo-Pacific and Caribbean.

Here, we address these problems using standardized sampling at seven localities in the eastern Indian Ocean, the western and the central Pacific, and the Caribbean (Fig. 1) and using DNA barcoding [11] to cluster individuals into operational taxonomic units (OTUs).

## Materials and Methods

### i. Sampling

New sampling locations included localities in the Indian Ocean (Ningaloo, western Australia), the western Pacific Ocean (Lizard and Heron Islands, Great Barrier Reef, Australia), the central Pacific (French Frigate Shoals (FFS), northwestern Hawaiian Islands) and the Caribbean (Bocas del Toro, Panama) (Fig. 1). Additionally, we included our previously published diversity results from the Northern Line Islands and Moorea (French Polynesia) in the central Pacific that were based on similar methods [12].

Similar-sized dead coral heads (diameter ∼30 cm, the “footprint” or planar reef area per head ∼π 15^2^ = 707 cm^2^) were used as standardized samples and were collected on the reef at a depth of 8 to 12 meters. In the Indo-West Pacific, dead coral heads from the family Pocilloporidae were collected; in the Caribbean (where pocilloporids do not occur), dead heads from three genera (*Eusmilia*, *Porites* and *Agaricia*) were collected to span as much diversity as possible. Dead heads were collected and processed following the method described in Plaisance et al. [12] with the exception that the heads were bagged before detaching from the bottom. The invertebrate community was extracted from the dead heads by breaking them into small pieces.

At FFS, no dead coral heads were collected to comply with the policies of the Papahanaumokuakea Marine National Monument; instead autonomous reef monitoring structures (ARMS) were deployed in 2006 and retrieved a year later. ARMS are small, long-term collecting devices designed to mimic, to some degree, the structural complexity of a coral reef. They consist of stacked layers of PVC with openings that allow organisms to settle or shelter within the structure (the “footprint” or planar reef area per ARMS is ∼529 cm^2^; http://www.pifsc.noaa.gov/cred/arms.php). During their retrieval, a mesh cage was placed around them to prevent escapes. The ARMS were disassembled and each layer was scanned carefully in order to collect all mobile invertebrates that had settled. In order to compare the effectiveness of dead coral heads and ARMS, both were collected at Heron Island.

For each reef sample (dead head or ARMS), all the crustaceans were sorted to morphospecies based on shape and color pattern. This was done conservatively (that is, if there were any doubts, organisms were considered to be distinct to minimize the chances of missing cryptic species). This method proved to be effective, as no cryptic diversity was recovered afterwards based on molecular information (Plaisance et al. [12]). For less common morphospecies, a tissue sample was collected from each individual; for morphospecies with ten or more individuals in a dead coral head or ARMS, ten individuals were haphazardly chosen for sampling. This same procedure was applied for each new head or ARMS sampled.

### ii. Molecular analysis

Tissue samples (most commonly a leg) were preserved for DNA analysis in 95% ethanol. Genomic DNA was extracted using standard proteinase-k digestion followed by phenol–chloroform extraction on the AutoGenprep 965 (Autogen). Standard PCR amplification using primers described in Folmer et al. [13] and automated sequencing techniques were used to sequence in both directions part of the mitochondrial COI gene used for DNA barcoding [11].

### iii. Statistical analysis

We used a 5% sequence dissimilarity threshold with the furthest neighbor clustering method for species discrimination because this value falls in a region where OTU numbers are relatively insensitive to the exact threshold chosen (see below and Plaisance et al. [12] for detailed justification of this threshold). The validity of this molecular threshold for the present dataset was tested by plotting the number of OTUs against different molecular thresholds to confirm the presence of a plateau at 5%.

Sequences were clustered into OTUs using MOTHUR [14]. Sequences were assigned into larger groups (e.g. decapods, brachyurans) based on field notes and closest barcode matches in GenBank. We employed ACE (Abundance-based Coverage Estimator) and Chao1 non-parametric estimators [15] to estimate total diversity, using either all samples for each locality (which varied from 6 to 23) or a subset of 6 samples randomized a thousand times (to eliminate sample size biases [16]). Both estimators use the number of rare species (for Chao I, the numbers of species occurring once and twice; for ACE, the number of species that occur from once to ten times) to adjust upward from the observed number of species. Individual-based rarefaction curves for each locality were also plotted. The Bray-Curtis similarity index was used to estimate the similarity in community composition within and between localities; to provide context, they were compared with the same indices calculated for reef slope communities found in supplemental Table 2 of Dornelas et al. [17]. To estimate the number of decapods potentially associated with coral reefs in the Ocean Biogeographic Information System (OBIS, www.iobis.org [18]), we searched for all taxa between 30°N and 30°S with minimum depth of 0 m and maximum depth of 40 m; double listings due to misspellings and errors associated with maximum depths listed as 0 rather than an empty cell were removed, but the number obtained remains an overestimate as some open water species were undoubtedly included.

## Results

**Figure 2:**
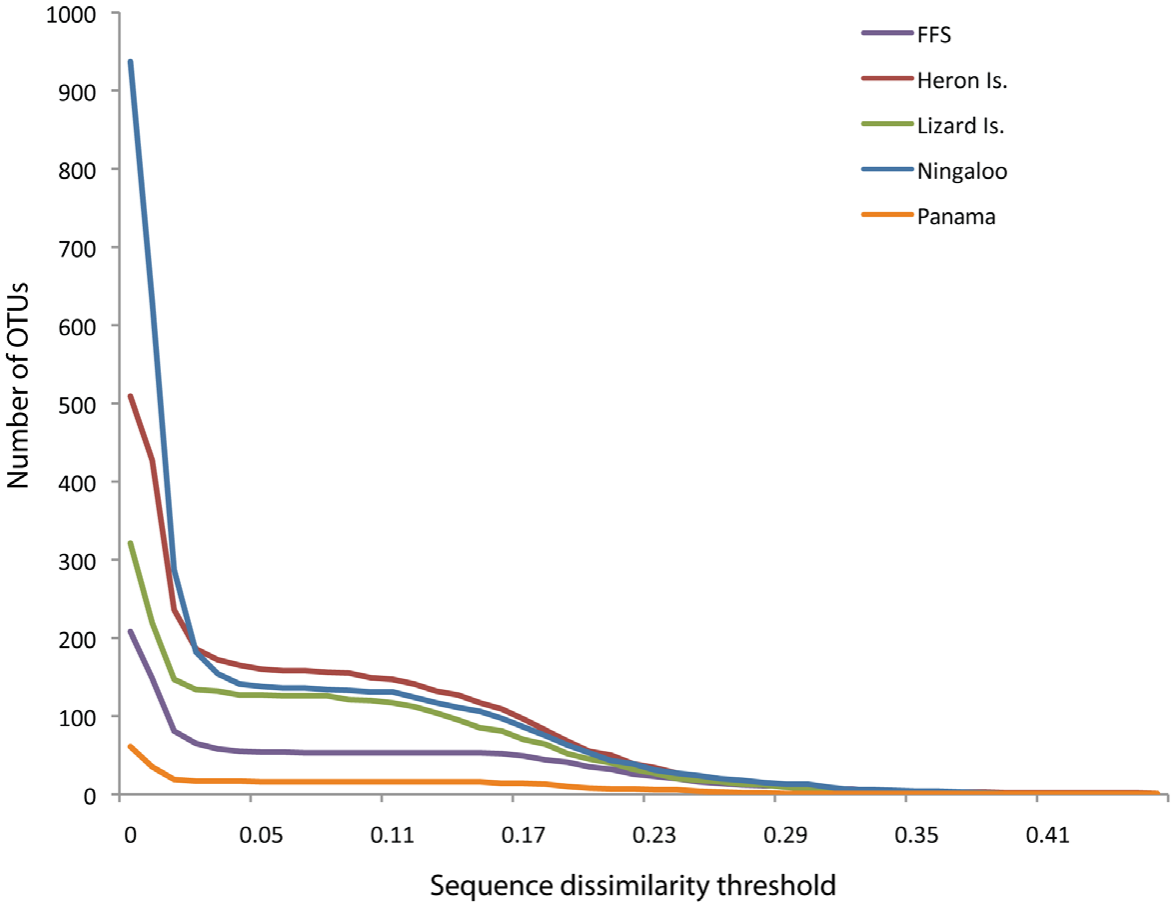
Step function analysis of the number of species found in sampling units (dead *Pocillopora* coral and ARMS) in the new localities investigated [French Frigate Shoals (FFS), Heron and Lizard Islands, Ningaloo and Panama] as a function of the cytochrome oxidase subunit I sequence dissimilarity threshold.

**Figure 3:**
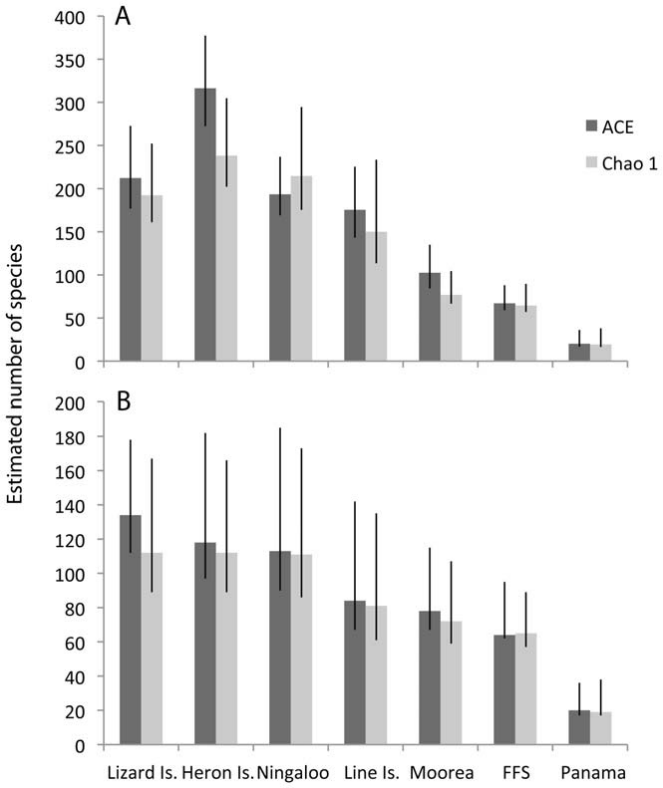
Estimated diversity values for seven sampled localities using the Abundance based Coverage Estimator (ACE) and Chao1 (+/− lower and higher bound of 95% confidence interval). A- Estimated diversity based on all samples. B- Comparable analysis restricted to six samples from each locality (in order to minimize the effect of different numbers of samples), randomized a thousand times. (FFS corresponds to French Frigate Shoals, Hawaii).

**Figure 4:**
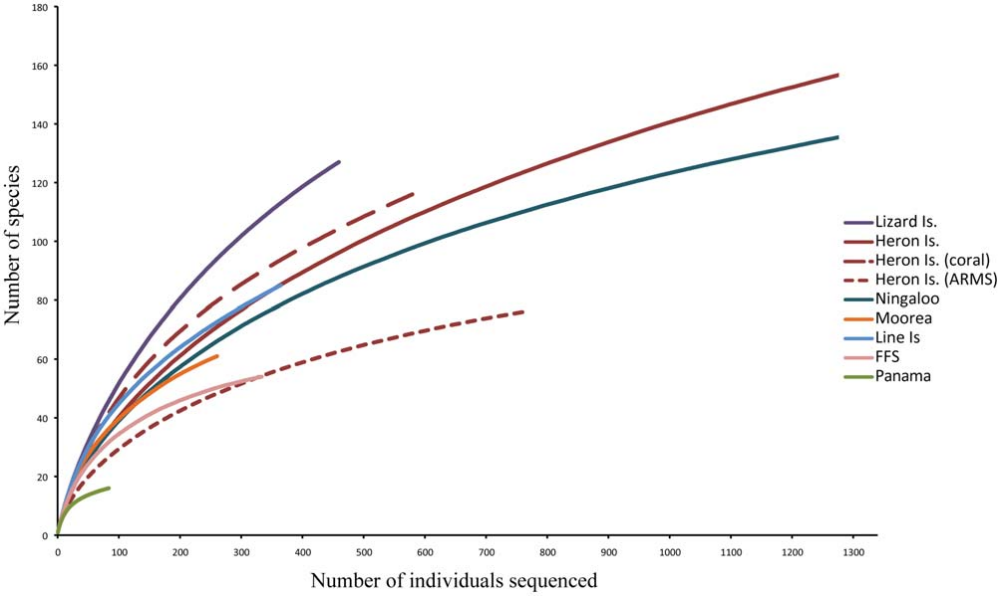
Individual-based rarefaction curves for the seven localities investigated (FFS corresponds to French Frigate Shoals, Hawaii) depicting the number of species recorded as a function of the number of individuals sequenced. For Heron Island, dead coral heads and artificial settlement structures (ARMS) are plotted both separately and combined.

In total, we analyzed DNA barcodes for 4182 crustaceans of which 3780 were new sequences (GenBank accession numbers: HM462477–HM466658). Overall, we identified 525 unique OTUs, 509 in the Indo-Pacific and 16 in the Caribbean (Table 1), using the criterion of 95% sequence similarity. This threshold generally corresponds with boundaries between morphologically defined species in crustaceans [19] and is located on a plateau where the numbers of OTUs are relatively insensitive to the precise cut-off value chosen [12] (e.g. between dissimilarities of 0.05 and 0.10, Fig. 2); this insensitivity suggests that most of the OTUs detected are also good biological species whether they be allopatric or sympatric. Only 3.2% of the OTUs matched other sequences deposited in GenBank at the 95% level (excluding matches with sequences previously deposited [12] that are included in this study). Of the 525 crustacean OTUs, 412 were decapods, and of these, 168 were brachyurans (true crabs); the remainder were amphipods, isopods, mysids, tanaids and stomatopods. Using Chao1 and ACE, the estimated total number of crustacean species ranged from 746–781 (Fig. 3), but these are likely to be underestimates because of the effect of the high numbers of singletons on even these estimators [16]. In particular, they underestimate true species diversity until the numbers of species sampled is ∼75% of total species richness, and one rule of thumb suggests this occurs when numbers of individuals sampled is ∼340–1100 times the number of species detected [16]. Rarefaction curves (Fig. 4) did not reach an asymptote at any site, indicating that a large number of species remain to be sampled, even where the sampling effort was highest.

**Table 1:** Sampling details and diversity results for each site and sites combined.

Locality	Lizard Island	Heron Island			Ningaloo	All IWP	N. Line Islands	Moorea	FFS	All CP	Panama	All Locations
Nature of sample	Dead Coral	Dead Coral	ARMS	Coral+ARMS	Dead Coral	Coral+ARMS	Dead Coral	Dead Coral	ARMS	Coral+ARMS	Dead Coral	Coral+ARMS
Number of samples	15	14	9	23	21	59	14	8	6	28	6	93
Estimated planar area (m^2^)	1.1	1.0	0.5	1.5	1.5	4.0	1.0	0.6	0.3	1.9	0.4	6.3
Number of sequences	460	580	760	1340	1338	3138	365	261	334	960	84	4182
Number of crustacean OTUs	127	116	76	160	138	355	85	61	54	180	16	525
Number of decapod OTUs	112	95	69	137	119	296	69	47	33	129	16	412
Number of brachyuran OTUs	50	44	14	50	56	122	36	24	12	58	5	168
Number (%) of crustacean singletons	40 (31.5)	32 (27.6)	17 (22.3)	49 (30.6)	41 (29.7)	130 (36.6)	34 (40)	17 (27.9)	12 (22.2)	64 (35.6)	5 (31.3)	199 (37.9)
Chao 1 estimate for full sample (randomized 6 sample subset)	192 (112)	202 (111)	94 (79)	238 (112)	215 (111)	528 (137)	150 (72)	77 (81)	65 (65)	267 (123)	19 (19)	781 (139)
ACE estimate for full sample (randomized 6 sample subset)	212 (134)	229 (114)	102 (86)	316 (118)	193 (113)	516 (149)	175 (84)	102 (78)	67 (67)	300 (128)	20 (20)	746 (152)

Presented are numbers of sampling units [dead coral heads or settlement devices (ARMS, see Methods)]; estimated total planar area sampled for each locality; the numbers of DNA sequences analyzed; the numbers of taxa (OTUs) for all crustaceans, all decapods, and brachyuran crabs; the numbers of crustacean singletons (see text) (percentages in parenthesis); and for crustaceans the ACE (Abundance-based Coverage Estimator) and Chao1 estimated diversity values [based both on all samples and just six samples per site (in parentheses, to eliminate biases caused by unequal sample numbers)]. The “IWP” and “CP” columns show the results for the localities of the Indo-West Pacific combined (Ningaloo, Lizard Island and Heron Island) and the Central Pacific combined [Moorea, Northern Line Islands and French Frigate Shoals (FFS), Hawaii] respectively.

The biogeographic patterns of diversity, the prevalence of rare species, and the lack of overlap between sites that we observed were consistent with previous studies [17], [20], [21], suggesting that the methods used provide a representative measure of species diversity. The three Indo-west Pacific (IWP) sites were more diverse than the three sites in the central Pacific (CP), which were more diverse than the Caribbean site (Table 1, Figs. 3A and B). Nearly 40% of the crustacean species (as defined by the 95% sequence similarity threshold) occurred just once, and only 16% were represented by more than ten individuals. Most species (81%) were found in only one locality, and values of the Bray-Curtis index of similarity (BCI, which ranges from 0 to 1) generally showed very little overlap between sites (Table 2). The two highest values were between the two sites from the central tropical Pacific (BCI = 0.12) and the two sites from the Great Barrier Reef (BCI = 0.24); the latter value is comparable to those observed for western Pacific coral communities from comparable depths (BCI = 0.20–0.26 for comparisons between Indonesia, Papua New Guinea and the Solomon Islands [17]).

**Table 2:** Bray-Curtis similarity indices for all pairwise comparisons between localities.

	Lizard Is	Heron Is	Ningaloo	Moorea	Line Is	FFS
Heron Is	0.24					
Ningaloo	0.042	0.053				
Moorea	0.041	0.022	0.021			
Line Is	0.019	0.004	0.016	0.118		
FFS	0.007	0.002	0.055	0.026	0.031	
Panama	0	0	0	0	0	0

FFS corresponds to French Frigate Shoals, Hawaii. The values were obtained by pooling all samples at each site and calculating the between site Bray-Curtis Index value, yielding one value for each pairwise by site comparison.

Artificial sampling devices gave somewhat lower numbers of species and rare species, but the patterns of diversity observed were as would be expected from longitudinal diversity gradients (Heron Island ARMS>French Frigate Shoals ARMS, Table 1). The similarity between artificial substrates and dead heads at Heron Island, where both were sampled, resembled that observed between dead heads at that site (pairwise between heads and artificial substrates mean BCI = 0.177, pairwise between heads mean BCI = 0.191). Moreover, the average Bray Curtis similarity index between pooled ARMS and pooled dead coral heads (0.41) is comparable to that observed between randomized pooled subsets of dead heads at Heron Island (0.53). Both of these values were within the range reported for mean within site similarity for corals of 0.359 to 0.667 by Dornelas et al. [17] and much higher than any between site similarity indices in our study (Table 2).

## Discussion

The combined planar area (i.e. basal area or footprint) of dead corals and artificial substrates sampled for this study was only ∼6.3 m^2^. Yet in this very limited sample, we found a total of 525 crustacean species; 412 of these were decapods, and of these 168 were brachyuran crabs, numbers that represent a surprisingly large percentage of numbers of species reported in global databases or much more intensive surveys. For example, for the comparatively better known brachyuran crabs, the number of species we detected in our samples is almost 80% of the number of described brachyuran species from all European seas [2] and 2.4% of the world's total (6978 species) based on the World Register of Marine Species (WoRMS [22]). Similarly, as of August 12^th^ 2010, there are only ∼1500 shallow water (less than 40 m depth) tropical (30°N-30°S) decapods recorded in the global Ocean Biogeographic Information System (OBIS [18]), a database increasingly used for marine biodiversity analyses [23].

Because the samples were taken from around the world, one cannot conclude that any single region or location would contain, for example, over 400 species of decapods in a sampled area of 6 m^2^ (although it is worth noting that none of our samples came from the most species-rich parts of the Indo-West Pacific). To further put these numbers in perspective, during a 2004 Philippines expedition, 74 scientists each working ∼30 days using hand, suction, trawl, dredge, and trap methods at 307 stations covering over 150 km^2^ ranging in depth from the intertidal to 130 m and including reefs, mangroves and soft bottoms collected ∼1200 decapod species [24]. Documented diversity gradients [25] suggest that a comparable effort (six person-years) would yield ∼900 decapod species from the Great Barrier Reef, yet we found 23% of that number (205 species) with a miniscule fraction of the effort and habitat diversity [two sites, combined collecting area of 2.1 m^2^ from a restricted depth range (8–12 m) and habitat type (forereef)].

Our finding of so many species in such a small total area and such restricted habitat types and depths, compared to the complexity and extent of coral reefs, suggests that tropical crustacean diversity (and likely the diversity of reefs overall) has been seriously under-detected, and by implication underestimated. Because dead coral heads and settling plates in shallow water are unlikely to host a fauna missed by traditional expeditionary methods, the most likely explanation for our findings is the systematic and intensive use of sensitive molecular methods to distinguish species. Although we also showed that very careful sorting of morphospecies by an experienced researcher was comparably effective at detecting cryptic species, this level of sorting may often not be done, and is very difficult to do accurately for samples collected and processed at different times.

Studies of microbial marine diversity have relied on molecular methods for more than two decades, yet discoveries of unexpectedly high diversity from small volumes of material continue to emerge [26]–[28] and no reliable global estimate is in sight. Comparable molecular analyses of standardized samples of small marine animals are in their infancy. For all such studies, estimates of regional and global diversity based on extrapolations from small samples have huge uncertainties. For example, the predicted global number of reef-associated brachyuran crab species based on our samples using a simple power-law model [Species = c(Area)^z^, where total area of our sample was 6.3 m^2^ and that of global coral reefs is 6×10^11^ m^2^ (1)], ranges from less than one third to nearly 600 times the total number (6978 [22]) of currently described brachyuran species for typical estimates of z (0.1–0.4; the commonly used z = 0.25 yields an estimate of over 13 times the number of described brachyurans). Moreover, z can vary with geographic scale [29] and alternatives to the power-law model may be superior in some cases [30].

Intensive surveys at single locations [24] are invaluable for interpreting the results of more limited sampling, but because they are so expensive, they cannot be repeated in many places. Thus, scalable, standardized and quasi-automated (e.g. molecular) approaches are needed to evaluate models, estimate parameters, and collect the amount of data required for meaningful global and regional diversity estimates and assessments of the effects of human impacts. This is particularly the case given the prevalence of rare species, implying that dense geographic sampling and large sample sizes per site [15] are required. Reefs are hard to sample, highly diverse, and seriously threatened [31], [32] but comparatively limited in area (<0.2% of the sea floor and ∼5% of tropical rain forest area [1]). This makes them a natural candidate for a comprehensive application of the quantitative and molecular sampling methods whose surprising effectiveness and ease of application we demonstrate here.
